# Gender differences in job quality and job satisfaction among doctors in rural western China

**DOI:** 10.1186/s12913-017-2786-y

**Published:** 2017-12-28

**Authors:** Yang Miao, Lingui Li, Ying Bian

**Affiliations:** 1 State Key Laboratory of Quality Research in Chinese Medicine, Institute of Chinese Medical Sciences, University of Macau, Taipa, Macau China; 2 The College of Management, Ningxia Medical University, Yinchuan, Ningxia China

**Keywords:** Gender differences, Job quality, Job satisfaction, Composite indicator, Rural western China

## Abstract

**Background:**

Few studies about gender differences in job quality and job satisfaction among medical professionals have been carried out in China. So the objectives of this study were to examine whether and to what extent gender differences existed in job quality and job satisfaction of doctors in rural western China.

**Methods:**

From 2009 to 2011, a total of 1472 doctors from 103 selected county-level health care facilities in rural western China were recruited into the study. Information about the doctors’ demographic characteristics, job quality, and job satisfaction was collected through a designed questionnaire. Besides examining gender differences in single dimensions of job quality and job satisfaction, principal component analysis was used to construct a composite job quality index to measure the differences in the comprehensive job quality, and exploratory factor analysis was applied to evaluate the differences in the overall job satisfaction. Chi-square test was used to calculate differences between proportions, and t-test was used to compare differences between means.

**Results:**

Among the doctors, there were 705 males and 767 females (ratio 1:1.09). Male doctors had significantly higher monthly salaries, longer working hours, more times of night shifts per month, longer continuous working hours, and longer years of service at current facilities, and marginally significantly higher hourly wage and longer years of service in current professions. However, female doctors showed greater overall job qualities. Significant and marginally significant gender differences were only found in satisfaction with remuneration compared to workload, the chance of promotion and working environment. But female showed greater satisfaction in the overall job satisfaction and the factor including sub-aspects of working environment, remuneration compared to workload, the chance of promotion, utilization of subjective initiative, and sense of achievement.

**Conclusions:**

Gender differences in job quality and job satisfaction did exist among doctors in rural western China. The participating female doctors were shown to have better job quality and greater job satisfaction.

**Electronic supplementary material:**

The online version of this article (doi: 10.1186/s12913-017-2786-y) contains supplementary material, which is available to authorized users.

## Background

Gender difference in labor market is a longstanding phenomenon, which far from disappearing in recent years, has been redefined [1]. As documented, the differences are mainly reflected in two domains, job quality and job satisfaction.

Job quality, which was traditionally understood as the wage level, has evolved into a multidimensional concept. The main components include socioeconomic security skills and training opportunities, working conditions, work-life balance, and promotion opportunities [2]. Each of these dimensions comprise several indicators, for example, socio-economic security is composed of indicators on wages and contracts; working conditions is represented by indicators on work intensity, long working hours, health risks, etc.

Previous studies have shown that gender differences may be reflected in many aspects of job quality, such as wages, type of contract, working hours, working conditions, skill and training opportunity, the chance of promotion, relationship with coworker and leaders and work-life balance [3, 4]. Women are more likely to be associated with employments with lower salaries, part-time positions, temporary contracts, and worse social protection; while in comparison, men are more prevalently to hold higher salaries, more chance of promotion, and upper category positions [4–6].

While job satisfaction, as a subjective notion, describing the level of contentment that employees feel about their jobs. A job that matches employees’ abilities and interests, increases employees’ engagement, and provides more opportunities of promotion is more easily to satisfy them [7]. Numerous studies have reported that women show greater job satisfaction than their male counterparts [8–11]. Analyses suggest that besides the systematic differences in working qualities experienced by women and men, different job expectations and values in job rewards cause the differences in job satisfaction between men and women. In some studies, the gender job satisfaction gap in favor of women has been attributed to their lower job expectations. The more or less pronounced disadvantage in the labor market, forces women to reduce their job expectations [12–14].

And some other studies indicate that men and women have different values concerning the import factors in their jobs. For men, they emphasize promotion prospects, pay and job security more highly than women; but for women, they are more concerned with good relations with managers and colleagues, the actual work itself and the hours of work [11, 12].

In the healthcare field, substantial progress toward gender working equality has been made in the past few decades. The increase in the proportion of women in medical schools denotes that women are receiving more equal educational and professional opportunities [15, 16]. However, gender disparities among the medical professionals still exist. The distribution of physicians across some specialties does not increase proportionally. Women physicians remain concentrated in a few specialties despite their increased representation in the profession, such as a vast majority of female gynecologists [17–20]. And still few women occupy senior positions in medicine [20, 21]. Statistics showed that in 2012 women physicians in the US earned 16% less than their male counterparts in the US [22]. While in terms of job satisfaction, only a few studies involve the discussion of gender differences, and the results are mixed. Some studies depict lower satisfaction among women than men [23, 24], and other studies report similar levels of satisfaction for both genders [23]. And furthermore, some studies claim gender has no effect on job satisfaction [25].

So far, despite the increasing attention on the phenomenon, few studies about gender influence on the job quality and job satisfaction of medical professionals have been carried out in China. Hence, the aim of this study is to examine gender differences in the job quality and job satisfaction of doctors in rural western China.

## Methods

### Sampling

Ten administrative areas in western China were selected to examine the job quality and job satisfaction of healthcare professionals, including Ningxia Hui Autonomous Region, Guangxi Zhuang Autonomous Region, Xinjiang Weiwuer Autonomous Region, Gansu Province, Shaanxi Province, Sichuan Province, Guizhou Province, Yunnan Province, Inner Mongolia and Tibet Autonomous Region.

In each administrative area, all counties were divided into three strata by GDP per capita (high, moderate, and low GDP per capita). And from each stratum, one county was randomly selected. Then the selected 30 counties were visited by the research teams from 2009 to the end of 2011. In each county, county-level health care facilities (usually including a general hospital, a traditional Chinese medicine hospital, a maternal and child care service center and a center of disease control) were investigated with medical professionals of various occupation groups (i.e. physician, nurse, pharmacist, and management staff). At each health care facility, 50 medical professionals were interviewed through a questionnaire.

In this paper, only gender differences among doctors were evaluated and discussed. And finally, a total of 1472 physicians (705 males and 767 females, ratio 1:1.09) from 103 selected county-level health care facilities were recruited.

### Questionnaire design

The questionnaire was consisted of two parts: the first part included questions about personal, professional and job characteristics of the participants (e.g. age, gender, education level, marital status, professional title, years of working, job engagement, job stability, payment, etc.); the second part was a series of 13 questions about job satisfaction with related single aspect, including satisfaction with job fulfillment, employee recognition, relationships with superiors, superiors’ decision-making ability, job security, opportunity to utilize skills and talent, institution policy implement, remuneration compared to workload, chance of promotion, utilization of subjective initiative, working environment, sense of achievement, and relationship with colleagues. For each question, the degree of satisfaction was rated using a 5-point Likert item, running from1 (very dissatisfied) to 5 (very satisfied).

With informed consent, all the participating doctors filled the questionnaire independently. And all the data were kept confidential just for this study and were not revealed or discussed with other people.

### Composite job quality index

Besides examining gender differences in each single dimension of job conditions, a composite job quality index (CJQI) was constructed to reflect the comprehensive job quality using principal component analysis (PCA).

The first step was to select individual variables of job quality (e.g. monthly salary, working hours, number of night shifts, etc.) that could be included in CJQI. Given that some of the variables were reversal indicators for CJQI, they needed to be recoded by negating. Normalization procedures were applied to eliminate differences in units of measurement. PCA was then used to calculate the weight of each variable, reduce the number of initial variables and obtain the principal components of the CJQI. The value of each principal component was equal to the linear sum of each normalized variable multiplying their factorial loads on the respective component and scaled by the square root of the component’s eigenvalue. And the weight of each principal component corresponded to the percentage of the variance it explained. Once weights were defined, the following step was a linear aggregation of the weighted sums of the values of the principal components.

At the end, to validate the constructed composite index, several PCAs were run with addition or subtraction of available indicators. And PCA yielding a higher KMO value indicated a more accurate model.

### Overall job satisfaction

To measure the overall job satisfaction of the doctors, exploratory factor analysis (EFA) was applied. First, a Cronbach’s alpha test was run to measure the internal consistency of the 13 Likert items to see whether they all reliably assessed job satisfaction. Then EFA was conducted to extract the appropriate number of factors, obtain all item loadings on each factor, and extract factor score coefficients of all items. The score of each factor was a summation of the factor score coefficient of each item multiplied to their corresponding scaled scores. The weight of each factor was equal to the percentage of the variance it explained. And the score for the overall job satisfaction was a linear aggregation of the weighted sums of all factor scores.

### Data collection and statistical analysis

Quantitative and qualitative data were collected through questionnaires completed by the participating physicians. All data were manually input and organized in Microsoft Excel 2013. IBM SPSS version 19.0 was employed for database assembling and statistical analysis.

Descriptive statistics for distribution, central tendency and dispersion were calculated. Chi-square test was used to calculate differences between proportions, and t-test was used to compare differences between means. Results with a 2-sided *p* value < 0.05 were considered statistically significant, and results with a 2-sided p value < 0.1 were considered marginally significant.

## Results

### Personal and professional characteristics

As exhibited in Table 1, participating male doctors were on average older than female (*p* = 0.002). Most of these physicians were married, and the majority owned college degree or above. No significant gender differences were found in their marital status and education levels. For both male and female doctors, resident position constituted the largest proportion of professional positions, and Chi-square showed significant gender differences existed in the distribution across professional positions (*p* = 0.009).

**Table 1:** Personal and professional characteristics of the doctors, by gender

		Female	Male
Age^**^	Mean (SD)	35.06 (8.16)	36.48 (9.03)
Marital status (%)	Married	79.35	77.71
	Single	20.65	22.29
Education level (%)	Master	1.18	1.85
	Bachelor	48.49	51.99
	College	38.50	35.33
	Secondary school	11.04	10.26
	Technical school	0.13	0.28
	High school	0.66	0.28
Professional title (%)^**^	Chief	0.40	0.87
	Associate chief	6.82	11.98
	Intermediate	26.07	27.56
	Resident	47.06	41.85
	Assistant	13.50	11.40
	TBD	6.15	6.35

^**^ *p* < 0.01

### Individual indicators of job quality

As shown in Table 2, male doctors had spent significantly longer time at current facilities (*p* = 0.007). Male doctors’ monthly salary, continuous working time, and number of night shifts per month were also significantly higher (*p* = 0.007, 0.013, and 0.023, respectively). Additionally, male doctors also had longer career length, longer weekly working hours and higher hourly wage, and the gender differences were marginally significant for the career length and hourly wage (*p* = 0.090 and 0.085, respectively), but not statistically significant for the weekly working hours.

**Table 2:** Individual job quality indicators of the doctors, by gender

	Female	Male
Monthly salary^**^	2666.04(1107.68)	2826.57(1163.11)
Weekly working hrs	57.05(18.17)	58.55(18.68)
Continuous working hrs^*^	21.22(17.00)	23.67(20.64)
Number of night shift^*^	4.82(4.34)	5.40(5.37)
Hourly wage^†^	12.54(5.65)	13.09(6.42)
Years at current facilities^**^	9.09(7.82)	10.24(8.32)
Total years of service^†^	13.31(9.16)	14.14(9.66)

^†^ *p* < 0.1, ^*^ *p* < 0.05, ^**^ *p* < 0.01

### Composite job quality index

When performing PCA, a correlation matrix was built as a prelim to check for the relationships among the original variables. Generally speaking, the correlations among variables should be not too low (*r* < 0.1) or too high (*r* > 0.9). As shown in Table 3, the correlations between the hourly wage, the weekly working hours, the continuous working time and the number of night shifts were moderate or low.

**Table 3:** Correlation matrix of original variables for CJQI

	Hourly wage	Weekly working hrs	Continuous working hrs	Number of night shifts
Hourly wage	1			
Weekly working hrs	-.484^a^	1		
Continuous working hrs	-.121^a^	.296^a^	1	
Number of night shift	-.232^a^	.442^a^	.313^a^	1

^a^Correlation is significant at the level 0.001

Validation and sampling adequacy showed that the best choice of variables for inclusion in the composite job quality index. With a value of 916.078 (*p* = 0.000), Bartlett’s test showed that the uncorrelated variable hypothesis could be rejected. And Kaiser-Meyer-Olkin measure of sampling adequacy was 0.642 (>0.6), justifying the use of this method in the present analysis.

To lower the number of original variables, PCA indicated that component 1, 2 and 3 were chosen to construct the CJQI since they three together explained around 89% of the total variability of the sample (Table 4). And variable participations in each component were shown in Table 5.

**Table 4:** Eigenvalues and percentage of variance explained by PCA for the CJQI

	Eigenvalue		
Component	Total	% of variance	Cumulative %
1	1.969	49.231	49.231
2	0.924	23.091	72.322
3	0.662	16.549	88.872
4	0.445	11.128	100.000

**Table 5:** Component matrix for the CJQI

Variables	Component		
	1	2	3
Hourly wage	.659	−.612	.271
Weekly working hrs	.832	−.194	−.020
Continuous working hrs	.721	.258	−.601
Number of night shift	.569	.667	.476

Note: Extraction method: principal component analysis

Then the CJQI was built as the following equation:

CJQI = 0.157^∗^Z_hourly wage_ − 0.271^∗^Z_weekly working hrs_ − 0.217^∗^Z_night shifts_ − 0.514^∗^Z_continuous working hrs_

Where Z_xi_ is the normalized variable.

Finally, to validate the composite index, several PCAs were run with hourly wage substituted by monthly salary, and with the addition of years of service at the current facility, or total years of service, or both of them. However, the PCAs yielded lower KMO values (0.627, 0.623, 0.634, 0.563), indicating less accurate models. This denoted that the CJQI as the above equation was a suitable model.

### Gender differences in CJQI

Descriptive analysis of CJQI for the participating doctors by gender is shown in Table 6. For the male doctors, the range of the CJQI was larger, and both the lower and upper limits of the CJQI were lower than those of their female counterparts. The average CJQI of the male doctors was significantly lower than that of the female (*p* = 0.022), indicating that the participating female doctors had comparatively higher job qualities.

**Table 6:** Descriptive analysis of the CJQI for the doctors, by gender

	Female	Male
Maximum	1.562	1.559
Minimum	−3.806	−7.077
Mean^*^	0.048	−0.052
SD	0.764	0.896

^*^ *p* < 0.05

### Single aspect of job satisfaction

The response rates for the survey about job satisfaction were about 98.87% for male and 98.18% for female.

Questions and doctors’ response distributions were listed in Table 7. As shown, for all the participating doctors, the satisfaction levels were relatively high in the relationship with colleagues, job fulfillment, job security, and opportunity to utilize skill and talents; and the levels were relatively low in remuneration compared to workload and chance of promotion. Gender differences were found significant for the distributions of satisfaction levels with remuneration compared to workload (*p* = 0.005), the chance of promotion (*p* = 0.026), and marginally significant for satisfaction with working environment (*p* = 0.058).

**Table 7:** Single aspect of job satisfaction for the physicians, by gender

		Level of satisfaction^a^ (%)				
		1	2	3	4	5
Job fulfillment	Female	1.99	4.24	38.68	39.47	15.50
	Male	1.72	6.17	40.03	36.44	15.64
Employee recognition	Female	4.11	7.42	51.39	27.55	9.27
	Male	4.88	10.47	46.34	27.55	10.76
Relationships with superiors	Female	3.58	9.67	44.11	33.25	9.40
	Male	6.17	9.76	44.76	28.84	10.47
Superiors’ decision-making ability	Female	4.50	7.95	42.91	34.17	10.33
	Male	6.17	8.46	43.47	30.13	11.76
Job security	Female	3.31	4.90	39.07	39.34	13.25
	Male	3.44	5.31	43.04	35.87	12.34
Opportunity to utilize skills and talents	Female	3.31	4.77	47.28	33.77	10.86
	Male	2.87	6.46	46.05	32.71	11.76
Institution policy implement	Female	4.77	9.40	49.54	27.81	8.48
	Male	5.45	11.19	50.07	24.68	8.61
Remuneration compared to workload^**^	Female	14.44	29.27	39.34	14.17	2.78
	Male	16.93	33.29	34.43	10.33	5.02
Chance of promotion^*^	Female	8.48	17.09	54.70	14.83	4.90
	Male	12.63	19.37	47.63	14.63	5.74
Utilization of subjective initiative	Female	4.11	11.52	53.25	24.11	7.02
	Male	6.31	14.20	48.49	24.39	6.60
Working environment^†^	Female	6.62	15.50	49.93	22.25	5.70
	Male	9.47	18.36	46.77	18.65	6.74
Sense of achievement	Female	4.24	11.39	45.30	29.67	9.40
	Male	7.17	11.19	44.76	27.83	9.04
Relationship with colleagues	Female	0.93	6.62	28.48	47.15	16.82
	Male	2.01	8.03	27.40	45.05	17.50

^a^1 = very dissatisfied, 2 = dissatisfied, 3 = neutral, 4 = satisfied, 5 = very satisfied

^†^ *p* < 0.1, ^*^ *p* < 0.05, ^**^ *p* < 0.01

### Overall job satisfaction

The resulted Cronbach’s alpha was 0.927, and a slight increase in alpha to 0.928 for the sample was achieved by eliminating item 13. It suggests that item 13- satisfaction with the relationship with colleagues should be removed from the following analysis, and the internal consistency of the rest 12 items were excellent to examine the overall job satisfaction.

Then the inter-correlation between the questions were checked and the results were shown in Table 8. None of the other items correlated too highly (*r* > 0.9) or too lowly (*r* < 0.1) with others, confirming that the EFA could be carried out with the rest 12 items. Then Kaiser-Meyer-Olkin measure of sampling adequacy was 0.933, and the result of Bartlett’s test was 12,017.788 (*p* = 0.000), justifying the sample was factorable. With a minimum eigenvalue criterion of 1.0 and varimax rotation, two non-trivial factors were extracted to explain about 68.485% of the variance of the 12 items (Table 9).

**Table 8:** Correlation matrix between 13 questions about job satisfaction

	Q1	Q2	Q3	Q4	Q5	Q6	Q7	S8	Q9	Q10	Q11	Q12	Q13
Q1	1												
Q2	.507^a^	1											
Q3	.437^a^	.745_a_	1										
Q4	.418^a^	.704^a^	.799^a^	1									
Q5	.502^a^	.651^a^	.683^a^	.716^a^	1								
Q6	.494^a^	.676^a^	.672^a^	.701^a^	.722^a^	1							
Q7	.408^a^	.653^a^	.698^a^	.721^a^	.662^a^	.710^a^	1						
Q8	.214^a^	.420^a^	.434^a^	.445^a^	.359^a^	.407^a^	.476^a^	1					
Q9	.282^a^	.559^a^	.531^a^	.552^a^	.472^a^	.505^a^	.549^a^	.655^a^	1				
Q10	.377^a^	.441^a^	.473^a^	.464^a^	.432^a^	.505^a^	.452^a^	.515^a^	.613^a^	1			
Q11	.302^a^	.404^a^	.441^a^	.438^a^	.384^a^	.399^a^	.433^a^	.594^a^	.601^a^	.620^a^	1		
Q12	.440^a^	.426^a^	.433^a^	.440^a^	.431^a^	.470^a^	.424^a^	.446^a^	.566^a^	.658^a^	.608^a^	1	
Q13	.319^a^	.270^a^	.275^a^	.284^a^	.293^a^	.324^a^	.231^a^	.263^a^	.395^a^	.553^a^	.405^a^	.566^a^	1

^a^Correlation is significant at the level 0.001

**Table 9:** Eigenvalues and percentage of variance explained by EFA for the overall job satisfaction

	Initial eigenvalue			Rotation sums of square loadings		
Component	Total	% of variance	Cumulative %	Total	% of variance	Cumulative %
1	6.801	56.675	56.675	4.698	39.149	39.149
2	1.417	11.810	68.485	3.520	29.336	68.485
3	0.851	7.091	75.576			
4	0.493	4.110	79.686			
5	0.407	3.395	83.081			
6	0.392	3.270	86.351			
7	0.335	2.789	89.141			
8	0.316	2.633	91.773			
9	0.298	2.483	94.257			
10	0.276	2.299	96.556			
11	0.228	1.897	98.453			
12	0.186	1.547	100.00			

Item participations into each factor and factor score coefficients were shown in Table 10. Seven items loaded onto factor 1, which accounted for the most variance 39.149%. The factor reported the satisfaction levels with superior’s decision-making ability, manners treated by the superior, institution policy implement, employee recognition, job security, job fulfillment, and opportunity to utilize skills and talents. The second factor explained 29.336% of the variance, with 5 items loaded on. This factor reported the satisfaction levels with working environment, remuneration compared to workload, the chance of promotion, utilization of subjective initiative, and sense of achievement.

**Table 10:** Rotated factor matrix and factor score coefficient matrix for overall job satisfaction^a,b,c^

	Factor^d^	
	1	2
Q1	.593 (.163)	.196 (−.066)
Q2	.804 (.215)	.290 (−.078)
Q3	.818 (.216)	.305 (−.075)
Q4	.823 (.216)	.310 (−.073)
Q5	.829 (.240)	.230 (−.114)
Q6	.808 (.214)	.299 (−.074)
Q7	.770 (.190)	.334 (−.047)
Q8	.234 (−.119)	.752 (.303)
Q9	.377 (−.067)	.750 (263)
Q10	.301 (−.101)	.775 (.295)
Q11	.208 (−.150)	.827 (.347)
Q12	.297 (−.092)	.737 (.278)

Note: ^a^Extraction method: principal component analysis. ^b^Rotation method: varimax with Kaiser normalization. ^c^Rotation converged in 3 iterations. ^d^Numbers in parentheses are the factor score coefficient for each item

Internal consistency for each factor was examined using Cronbach’s alpha. The alphas were 0.924 and 0.876 for factor 1 and 2, respectively. And no substantial increases in alpha for any of the factor when eliminating any items.

### Gender differences in the overall job satisfaction and the respective factors

As shown in Table 11, for both genders, the satisfaction levels for factor 1, factor 2 and the overall satisfaction were all from 1 to 5. While the average scores indicated that compared to male doctors, female ones had relatively high satisfaction levels in all the three dimensions. And the gender differences were found statistically significant for satisfaction with the second factor, and marginally significant for the overall satisfaction.

**Table 11:** Descriptive analysis of the factors and the overall job satisfaction, by gender

	Score	Female	Male
Factor 1	Maximum	5	1
	Minimum	1	1
	Mean	3.30	3.25
	SD	0.68	0.75
Factor 2^*^	Maximum	5	5
	Minimum	1	1
	Mean	3.12	3.04
	SD	0.67	0.77
Overall satisfaction^†^	Maximum	5	5
	Minimum	1	1
	Mean	3.22	3.16
	SD	0.66	0.74

^†^ *p* < 0.1, ^*^ *p* < 0.05

## Discussion

The results presented above indicate that among the doctors in this study significant gender differences did exist in job quality and job satisfaction.

### Findings

Among the 1472 participating doctors, there were slightly more females. No gender differences were found in their educational background. However, significant gender differences existed in the professional position distribution. More male held chief and associate chief positions, while more women held resident and assistant resident positions. The differences denote the possible existence of vertical segregation among the physicians. However, male doctor’s longer duration in this career and in current facilities indicates more experience of male might be a reason for the segregation.

With respect to individual job quality characteristics, male doctors in this study had more times of night shifts and longer continuous working hours, which might be due to the biological differences between male and female, that males could afford more physically demanding work. As a stressful job, doctors consistently experience high intensity of work, conflicting time demands, and heavy professional responsibility. So a qualified doctor needs to be not only encyclopedically medical knowledgeable but physically strong. Another explanation for the differences might be that women tend and are supposed to take more responsibility and spend more time in the family, so they might prefer specialties with more flexibility and less working time [26–29]. However, in the current study, specialties of the physicians were not available for analysis. Furthermore, compared to female doctors, males had higher monthly salaries and hourly wages. However, their weekly working hours were not significantly different. It means that male’s higher monthly salary and hourly wage are due to their more overtime pay, of which the rate is usually higher than the regular working hour pay rate.

In the resulted CJQI, the indicator with the greatest weight was the working hours, and the one with the smallest weight was the hourly wage, which suggests that the working hours is the most significant factor affecting the comprehensive job quality of these doctors, while hourly wage is the least significant factor. The results are inconsistent with some previous studies, which showed that pay was the most important problem affecting employee’s job quality, since it not only symbolized personal value and success in work life but represented job and life security [30]. Consequently, although the male doctors had comparatively higher hourly wages, their comprehensive job quality was lower than the females.

Among the 13 single aspects of job satisfaction, significant gender differences were found in satisfaction with the remuneration compared to workload, chance of promotion and working environment. Compared to female, more proportions of male felt dissatisfied with the three aspects. This finding is partially consistent with previous analysis of single job quality indicators that the male doctors had marginally significantly higher hourly wage but significantly more times of night shift and longer working hours.

Furthermore, marginally significant gender differences and significant gender differences were found in the overall job satisfaction and satisfaction with the personal factor, respectively. This indicates that the female doctors showed greater job satisfaction, especially greater satisfaction with working environment, remuneration compared to workload, chance of promotion, utilization of subjective initiative, and sense of achievement. The findings are also in line with the previous analysis for job quality and single aspects of job satisfaction. Other explanation for female’s greater satisfaction might be that male and female value job characteristics differently. As a general rule, female are socialized to have lower expectations about jobs [8, 12, 26, 31, 32], and place less value on remuneration and promotion [8, 12, 33], so they are more easily satisfied.

### Limitations

Firstly, this study only examined a limited number of objective job characteristics, which mainly focused on wages and working time. However, sociologists and organizational psychologists have perceived job quality through a broader lens including employee’s well-being, work-life balance, job autonomy, and personal development. And within the framework of the European Employment Strategy, 10 groups of indicators have been defined to monitor employment quality, including intrinsic job quality; skills, life-long learning and career development; gender equality; health and safety at work; flexibility and security; inclusion and access to the labor market; work organization and work-life balance; social dialogue and worker involvement; diversity and non-discrimination; overall economic performance and productivity [34]. So, further studies are needed to include more variables regarding job quality, such as benefits, pension and insurance, training opportunity, and job flexibility etc.

Secondly, earlier studies have shown that male and female may place different values on difference job characteristics when determining the overall job quality and job satisfaction. However, in this study, both PCA and EFA analysis are methods used when no consensus about the relative importance of the original variables exists. So in the future survey, the relative importance of job characteristics needs to be included in survey questions when evaluating job quality and job satisfaction.

## Conclusions

Gender differences in job quality and job satisfaction did exist among doctors in county-level hospitals of rural western China. The participating female doctors were shown to have better job quality and greater job satisfaction. Future studies are needed to replicate the results with a wider range of participants and more indicators of job quality and job satisfaction, and identify the underlying reasons for the gender differences.

## Additional file

Additional file 1: Organized original database for analization. (XLSX 195 kb)
